# The Influence of Different Operation Conditions on the Treatment of Mariculture Wastewater by the Combined System of Anoxic Filter and Membrane Bioreactor

**DOI:** 10.3390/membranes11100729

**Published:** 2021-09-24

**Authors:** Yi Ding, Zhansheng Guo, Binyu Ma, Fang Wang, Hong You, Junxue Mei, Xuguang Hou, Zhenlin Liang, Zhipeng Li, Chao Jin

**Affiliations:** 1 Marine College, Shandong University, Weihai 264209, China; dingyits@126.com (Y.D.); guozhansheng@sdu.edu.cn (Z.G.); meijunxue@sdu.edu.cn (J.M.); richardhoukk@163.com (X.H.); liangzhenlin@sdu.edu.cn (Z.L.); 2 State Key Laboratory of Urban Water Resources and Water Environment, School of Marine Science and Technology, Harbin Institute of Technology at Weihai, Weihai 264200, China; a958444903@foxmail.com (B.M.); wf17612230450@163.com (F.W.); youhong@hit.edu.cn (H.Y.); 3 School of Environmental Science and Engineering, Sun Yat-Sen University, Guangzhou 510275, China

**Keywords:** mariculture wastewater, anoxic filter, membrane bioreactor, operation conditions, pollutants removal efficiency

## Abstract

The mariculture wastewater treatment performance for the combined system of anoxic filter and membrane bioreactor (AF-MBR) was investigated under different hydraulic retention times (HRTs), influent alkalinity, and influent ammonia nitrogen load. The results showed that the removal efficiencies of TOC and total nitrogen were slightly better at the HRT of 8 h than at other HRTs, and the phosphate removal efficiency decreased with the increase of HRT. With the increase of influent alkalinity, the removal of TOC and phosphate did not change significantly. With the increase of influent alkalinity from 300 mg/L to 500 mg/L, the total nitrogen removal efficiency of AF-MBR was improved, but the change of the removal efficiency was not obvious when the alkalinity increased from 500 mg/L to 600 mg/L. When the influent concentration of ammonia nitrogen varied from 20 mg/L to 50 mg/L, the removal efficiencies of TOC, phosphate, and total nitrogen by AF-MBR were stable. An interesting finding was that in all the different operation conditions examined, the treatment efficiency of AF-MBR was always better than that of the control MBR. The concentrations of NO_3_^−^-N in AF-MBR were relatively low, whereas NO_3_^−^-N accumulated in the control MBR. The reason was that the microorganisms attached to the carrier and remained fixed in the aerobic and anoxic spaces, so that there was a gradual enrichment of bacteria characterized by slow growth in a high-salt environment. In addition, the microorganisms could gather and grow on the carrier forming a biofilm with higher activity, a richer and more stable population, and enhanced ability to resist a load impact.

## 1. Introduction

Driven by the world population growth demand, mariculture industry had developed rapidly [1,2]. A large amount of wastewater produced by the marine aquaculture industry, characterized by high salinity and a large amount of suspended solids, nitrogenous compounds, and organic matter, is directly discharged into the environment, which would cause great harm to the environment [3]. Marine aquaculture wastewater could be treated by physical chemical or biological methods, among which biological methods have the unique advantage of reducing costs [4,5,6]. The membrane bioreactor (MBR) has been used in the field of high salinity wastewater treatment [7,8,9]. The high separation capacity of a membrane enables to completely retain the biomass, which is conducive to biomass adapting to a specific environment. On the basis of these advantages, MBR has great potential for high-salinity wastewater treatment [10].

The application of MBR under high-salinity conditions might exacerbate the problem of MBR membrane fouling [11,12]. High salinity destroys the multivalent cation bridge between the extracellular polymeric substance of this system in the sludge matrix [13,14], reduces protozoa and filamentous bacteria, and worsens the characteristics of the sludge, causing increased membrane fouling [15]. Sun et al. verified that high salinity also caused the production of extracellular polymeric substances through cell secretion and autolysis, which were considered to be the main cause of membrane pollution [16]. The characteristics of high salinity and high ammonia nitrogen limit the wide application of MBR in the treatment of mariculture wastewater [17].

The application of a biological carrier caused most of the sludge in the system to adhere to the carrier and reduced the direct contact of the sludge with the membrane surface, greatly decreasing the membrane fouling potential of the system [18]. In addition, the abundant biofilm on the carrier could adsorb and degrade fine suspended solids, colloidal particles, and macromolecular organics, avoiding their adsorption and deposition on the membrane surface or membrane pores [19]. There have been many reports on the effect of a biological carrier in alleviating membrane fouling in MBR in recent years [20,21]. Most of the research results showed that the introduction of a biological carrier could improve the characteristics of the sludge mixture in MBR, allowing for membrane fouling mitigation [22]. The membrane fouling mitigation mechanism of biological carriers could be divided into two categories: on the one hand, biological carriers, especially moving carriers, exert a mechanical scouring effect on the membrane surface [23]; on the other hand, the carrier improves the characteristics of the sludge mixture through biological immobilization and stabilization [24]. In recent years, many researchers carried out research on the membrane pollution mechanism of different forms of biological carriers in MBR, which laid a foundation for the application of biological carriers to alleviate MBR membrane pollution [23,25].

The carrier provides a stable growth environment for microorganisms, could promote the enrichment of some slow-growing strains, increase species diversity, and improve the decontamination efficiency of the system. Randall and Sen conducted a systematic study on the enhanced biological nitrogen removal process with a fixed carrier, and the results showed that the application of a carrier increased the nitrification efficiency per unit volume by 225% and the denitrification efficiency by 30–88% [23]. Deng et al. studied the enhancement effect of a new sponge carrier on the performance of MBR and showed that the application of a sponge carrier significantly improved the organic matter and ammonia nitrogen removal efficiency of the MBR system [26]. Nguyen et al. developed a new submerged sponge carrier MBR system and investigated its performance in treating domestic wastewater, and the results showed that the application of a biological carrier in MBR significantly improved the nitrogen and phosphorus removal efficiency of the system [27]. Leyva Diaz et al. systematically investigated the organic matter and nutrient removal efficiency of a biological carrier in MBR and showed that the removal efficiency of TN (67.34%) and TP (50.65%) of the MBR system with a biological carrier was slightly higher than that of the control system [28]. In addition, due to its good biological stabilization and immobilization, a biological carrier could promote the acclimation and enrichment of microorganisms in a high-salt environment, so potentially allowing the treatment of high-salt wastewater [29,30].

In conclusion, biological carrier technology is an effective means to increase MBR removal efficiency and control membrane fouling and has the potential to be applied in high-salt wastewater treatment. In a previous study, a new anoxic biofilm–moving bed MBR system was established for the treatment of high-salt mariculture wastewater. During the establishment of the combined system, the treatment performance and membrane fouling mitigation mechanism of the combined system were systematically studied, and a high nitrogen removal efficiency as well as a significant membrane fouling control in the high-salt environment of mariculture wastewater were realized [31].

Changes in the process operation parameters and influent water quality of the combined system may have a significant impact on the stable operation and treatment efficiency of the system. For the system used for biological treatments, the operation parameters (hydraulic retention time (HRT)) and water quality conditions (influent C/N ratio, alkalinity, etc.) could directly affect the operation performance of the system. Johir et al. showed that HRT was directly related to the ammonia nitrogen removal rate [32]. Viero et al. showed that a longer HRT could reduce sludge production and the aeration operation cost of MBR [33]. Chiu et al. showed that the C/N ratio is an important water quality parameter affecting the efficiency of biological nitrification and denitrification, and a low C/N ratio would reduce the efficiency of denitrification due to the lack of a carbon source [34]. Hu et al. found that alkalinity was closely related to the treatment efficiency of the MBR system. Increasing alkalinity could improve the removal rate of COD and ammonia nitrogen [35].

The effect of the operation parameters on the system’s treatment efficiency was rarely studied in relation to high-salinity mariculture wastewater. Therefore, in this study, the treatment efficiency of a system consisting of an anoxic filter and a membrane bioreactor (AF-MBR) for mariculture wastewater was investigated, changing HRT, influent alkalinity, and influent ammonia nitrogen load. The study of the effect of different operation conditions on the treatment of mariculture wastewater by AF-MBR can provide a theoretical basis for the subsequent application of AF-MBR in the treatment of high-salinity wastewater, which has practical value.

## 2. Materials and Methods

### 2.1. The AF-MBR System

**Figure 1:**
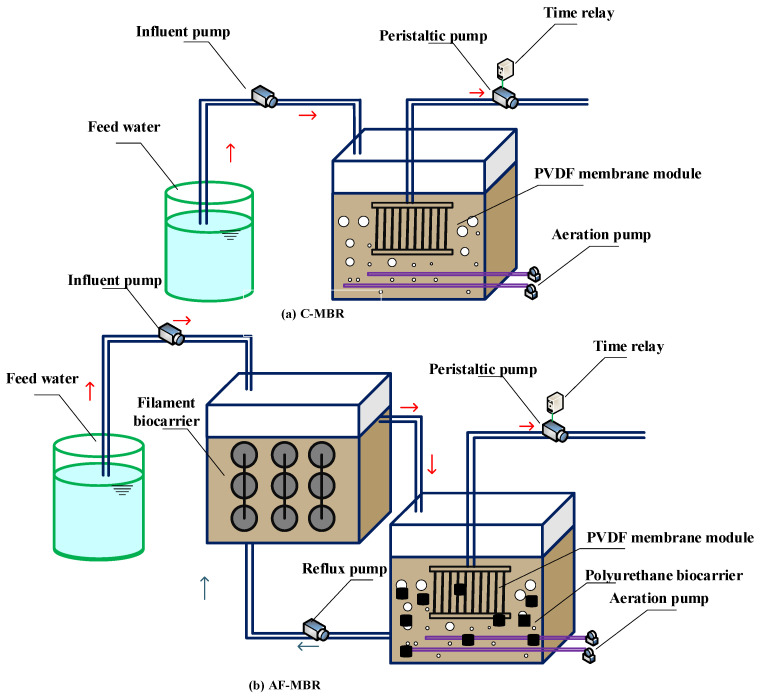
C-MBR (**a**) and AF-MBR (**b**) used in the experiment.

In this experiment, two sets of experimental devices were used, namely, C-MBR (control MBR) and AF-MBR, as shown in Figure 1. The combined AF-MBR system is made of plexiglass with an anoxic filter bioreactor and an MBR. The AF-MBR system mainly includes an influent pump and pipeline, an anoxic filter reactor, a membrane bioreactor, an aeration system, and effluent peristaltic pump and pipeline. The working volume of each reactor was 11 L, and it was designed as a square reactor with the size of 30 cm × 25 cm × 17 cm.

### 2.2. Operation of AF-MBR

In this experiment, the influent of AF-MBR system simulated mariculture wastewater [31]. Crude starch was used as the organic carbon source; ammonium chloride (NH_4_Cl), sodium dihydrogen phosphate (NaH_2_PO_4_), and disodium hydrogen phosphate (NaH_2_PO_4_) were used as nutrients. Seawater collected from Huang Hai (Shidao, Weihai, Shandong, China) was used to produce the mariculture wastewater. The seawater used in the preparation of mariculture wastewater was naturally purified seawater filtered through micropores. The inoculated sludge was collected from the secondary sedimentation tank of a wastewater treatment plant in Weihai (Shandong, China), which was acclimatized for a long time with simulated mariculture wastewater.

In this paper, the efficiency of AF-MBR and control MBR in the treatment of mariculture wastewater was studied under different operation parameters, such as different HRTs, alkalinity, and ammonia load. With the influent ammonia nitrogen concentration of 50 mg/L, the influence of HRT and influent alkalinity on the AF-MBR treatment performance was investigated.

### 2.3. Analytical Methods

The water samples were taken from influent and effluent. Each water sample was centrifuged at 5000 rpm for 5 min. After centrifugation, the water sample was filtered using a 0.45 μm membrane to obtain the filtrate to be measured. In this study, pH, alkalinity, nitrogen concentration, and phosphorus concentration were determined according to the method for water and wastewater monitoring for water quality determination [36]. Total nitrogen and TOC were measured by a TOC instrument (TOC-5000A, Shimadzu, Kyoto, Japan).

## 3. Results

### 3.1. Effect of Different HRTs on AF-MBR Treatment Performance

When using biological methods to treat sewage, the HRT is a very important parameter [37,38]. If the HRT is too short, the microorganisms do not have enough time to complete the process of nitrogen and phosphorus removal. Therefore, the removal efficiency of nitrogen and phosphorus is not good. If the HRT is too long, the running time would be too long, increasing the cost of the process. Therefore, it is necessary to determine the optimal HRT for process operation.

In this experiment, four HRTs (6 h, 8 h, 10 h, and 12 h) were set up to investigate the removal efficiency of TOC, phosphate, and total nitrogen by AF-MBR, and the concentrations of ammonia nitrogen, nitrate nitrogen, and nitrite nitrogen in AF-MBR and C-MBR were determined to evaluate the denitrification mechanism.

#### 3.1.1. Influence of Different HRTs on TOC Removal Efficiency

**Figure 2:**
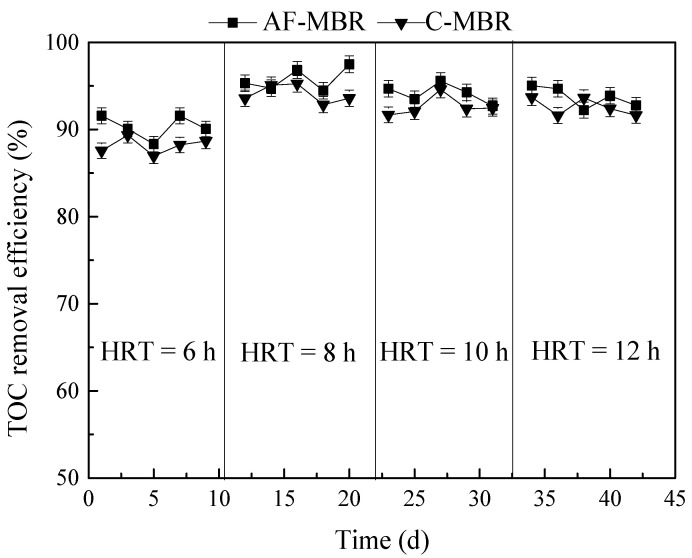
Influence of different HRTs on TOC removal efficiency.

The treatment performance of TOC for AF-MBR and C-MBR is shown in Figure 2. The MBR process requires a high biomass density and is based on the filtration and interception functions of membrane module; it can efficiently remove organic matter. It can be seen in Figure 2 that at the HRT of 6 h, the TOC removal efficiency of C-MBR was between 86.95% and 89.35%, and that of AF-MBR was between 88.32% and 91.57%. At the HRT of 8 h, the TOC removal efficiencies of AF-MBR and C-MBR were both higher than 90%. It was shown that the TOC removal efficiency was a little better at the HRT of 8 h than at other HRTs. The longer the HRT, the lower the concentration of organic matter in later stages of the process. Therefore, when increasing the HRT from 8 h to 12 h, it could be seen that the removal efficiency of TOC by AF-MBR and C-MBR was little affected by the HRT. This could be attributed to membrane retention and enhanced biodegradation (owing to increased biomass and acclimated microbial community) [39]. During the whole process of HRT change, the treatment efficiency of AF-MBR was always slightly higher than that of C-MBR. AF-MBR had a better organic load-bearing capacity, which was related to the application of a biological carrier to strengthen the biochemical decontamination efficiency of the system [26].

#### 3.1.2. Influence of Different HRTs on Phosphate Removal Efficiency

**Figure 3:**
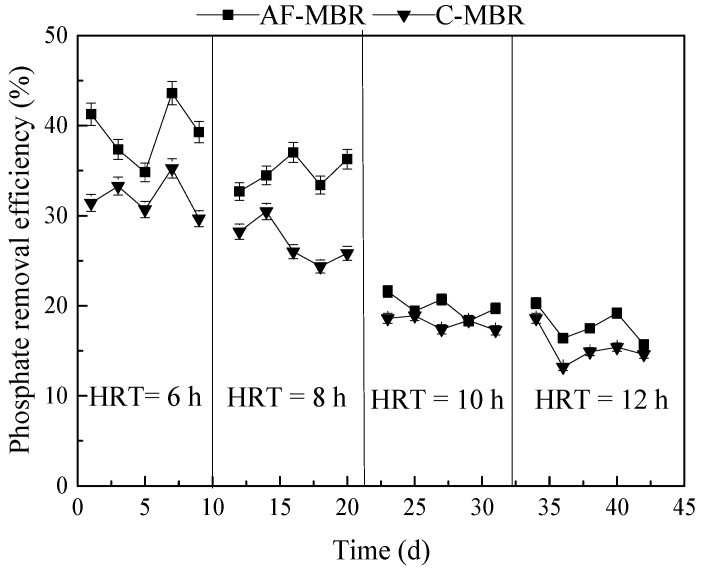
Influence of different HRTs on phosphate removal efficiency.

The treatment performance of AF-MBR and C-MBR in phosphate removal is shown in Figure 3. It can be seen from Figure 3 that, with the increase of HRT, the phosphate removal efficiency of AF-MBR decreased from 43.61% to about 15.70%, while that of C-MBR decreased from 35.26% to about 13.20%. The principle of using microorganisms to remove phosphate in wastewater is that in a system with an alternating operation of anaerobic-aerobic conditions and anaerobic-anoxic conditions, the concentration of phosphate in the aerobic and anoxic conditions is greatly reduced due to the anaerobic release of phosphorus by phosphorus-accumulating bacteria and the absorption of phosphorus by aerobic (or anoxic) bacteria; a large amount of phosphorus-rich sludge is discharged to remove phosphorus from the wastewater [40,41]. In C-MBR, there was no alternation of aerobic and anoxic operations, so the phosphorus-accumulating bacteria could not complete the aerobic phosphorus absorption and anaerobic phosphorus release processes. The growth of microorganisms was limited to a certain extent under high salt conditions; therefore, the removal efficiency of phosphate was not high in this experiment, due to less sludge discharge.

It was shown that the removal of biological phosphorus was better with lower HRT. With the increase of HRT, the concentration of organic matter in the later stages of the process was lower. Lowering the concentration of organic matter did not increase sludge concentration. Because phosphorus was removed through sludge wasting [42], the absorption of phosphorus by microorganisms was reduced, resulting in a decreased removal efficiency of phosphate. Due to the limitation of sludge discharge during the operation of AF-MBR, the removal efficiency of phosphate was not high but was better than that obtained with C-MBR.

#### 3.1.3. Influence of Different HRTs on Nitrogen Removal Efficiency

**Figure 4:**
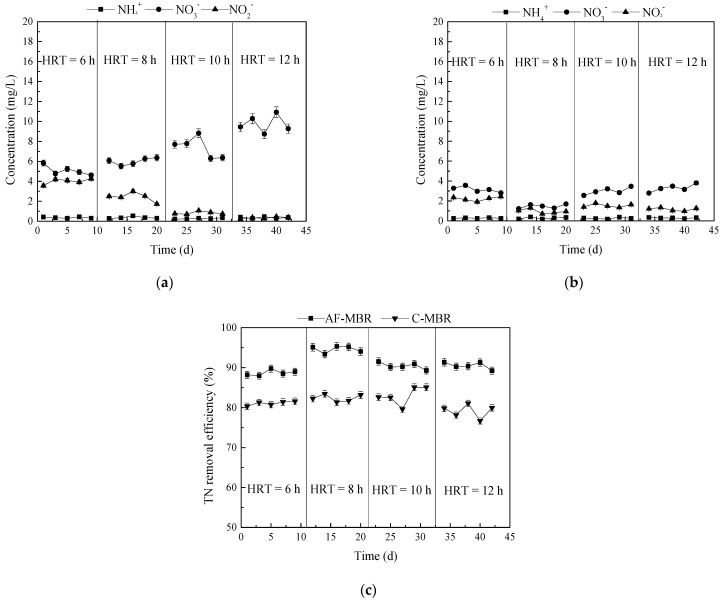
Effect of different HRTs on nitrogen treatment. (**a**) Changes of NH_4_^+^-N, NO_2_^−^-N, and NO_3_^−^-N concentrations in C-MBR; (**b**) changes of NH_4_^+^-N, NO_2_^−^-N, and NO_3_^−^-N concentrations in AF-MBR; (**c**) TN removal efficiency.

The changes of NH_4_^+^-N, NO_2_^−^-N, and NO_3_^−^-N in C-MBR and AF-MBR at different HRTs are shown in Figure 4a,b. In C-MBR, with the extension of HRT, the concentration of NH_4_^+^-N was maintained at a very low level, while the concentration of NO_2_^−^-N decreased, and the concentration of NO_3_^−^-N increased. It can be seen that the change of HRT had little effect on the concentration of various kinds of nitrogen in AF-MBR, and the concentrations of NH_4_^+^-N, NO_2_^−^-N, and NO_3_^−^-N were very low. The changes of total nitrogen in the AF-MBR and C-MBR systems at different HRTs are shown in Figure 4c. It can be seen that, with the extension of HRT, the removal efficiency of AF-MBR and C-MBR did not show obvious changes; the efficiency was slightly better at the HRT of 8 h than at other HRTs. It was reported that, as the HRT was reduced from 24 h to 12 h, the total nitrogen removal efficiency improved from 68.5% to 80.4%, but there was no further improvement when the HRT decreased to 6 h [42]. In this study, the reason for the better removal efficiency at the HRT of 8 h is that a highly saline condition may inhibit microbial activity at lower HRTs, and microbial acclimatization to the saline environment of the bioreactor could recover the biological performance at longer HRTs.

In the whole process, the removal efficiency of total nitrogen by AF-MBR was always better than that of C-MBR. The biological process of nitrogen removal mainly includes nitrification under aerobic conditions and denitrification under anoxic conditions, so that nitrogen-containing compounds are finally converted into N_2_ and removed from the system [43]. For C-MBR, there was dissolved oxygen in the reactor, so the nitrification reaction was carried out, and the concentration of NO_3_^−^-N increased with the extension of HRT. However, the nitrogen still existed in the form of NO_3_^−^-N in the system and was not removed efficiently. AF-MBR was operated alternately in the aerobic and anoxic conditions through reflux. In AF-MBR under aerobic conditions, NH_4_^+^-N in wastewater was converted into NO_2_^−^-N and NO_3_^−^-N by nitrification, and NO_2_^−^-N and NO_3_^−^-N entering the anoxic filter were removed by denitrification of through reflux.

This was mainly due to the application of a carrier in the combined system. On the one hand, the carrier provided a stable growth space for microorganisms. Microorganisms attached to the carrier and fixed in aerobic and anoxic spaces, so that nitrifying bacteria and denitrifying bacteria with slow growth in high-salt environment could be gradually enriched. In addition, microorganisms could gather and grow on the carrier to form a biofilm structure with higher activity, richer population, and increased stability, enhancing its ability to resist the impact of a load.

### 3.2. Effect of Different Influent Alkalinity Values on AF-MBR Treatment Performance

In the process parameters of biological wastewater treatment, the alkalinity of the influent is also a very important factor. The microorganisms need enough alkalinity as electron receptor and also regulate the pH value of water [41].

Since the main nitrogen removal mechanisms for wastewater treatment are nitrification and denitrification, the demand for alkalinity is continuous during the whole nitrification and denitrification processes. The alkalinity of 300, 400, 500, and 600 mg/L was set at the HRT of 8 h, and the independent C-MBR was used as the control. The treatment efficiency of AF-MBR for TOC, phosphate, and total nitrogen was investigated, and the concentrations of NH_4_^+^-N, NO_2_^−^-N, and NO_3_^−^-N in C-MBR and AF-MBR were determined to explore the nitrogen removal mechanism.

#### 3.2.1. Influence of Different Influent Alkalinity Values on TOC Removal Efficiency

**Figure 5:**
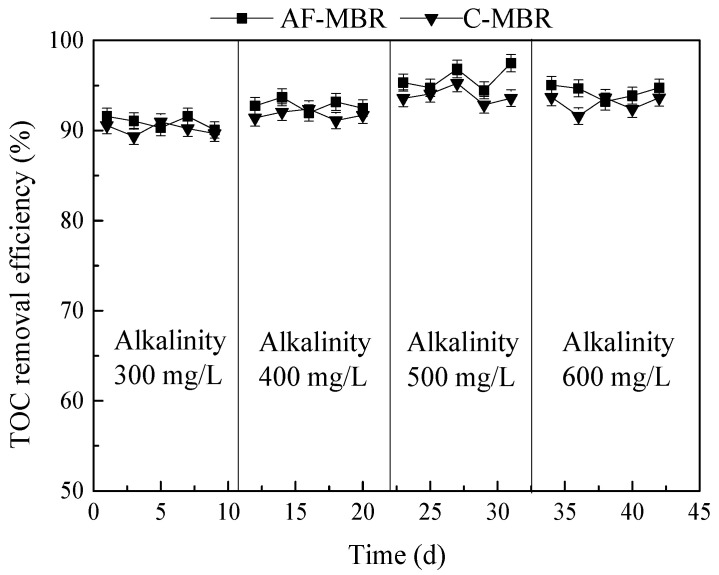
Influence of different influent alkalinity values on TOC removal efficiency.

The treatment efficiency of AF-MBR and C-MBR in TOC removal under different influent alkalinity is shown in Figure 5. It can be seen that the change of influent alkalinity had little effect on the removal efficiency of TOC. The removal of organic matter in wastewater mainly depends on the existence of a large number of microorganisms in the reactor. The change of alkalinity has little effect on the utilization of organic matter by the microorganisms. Therefore, the TOC treatment efficiency of AF-MBR or C-MBR was higher than 89.35%, and the treatment efficiency of AF-MBR was slightly better than that of C-MBR. When the alkalinity of the influent was 500 mg/L, the treatment efficiency in TOC removal was a little better than for other values of alkalinity.

It was reported that when the alkalinity is low, the pH of the system would decrease significantly as the reaction proceeds, which will affect microbial activity and reduce the treatment efficiency of the system [44]. However, in this study, even if the applied alkalinity was as low as 300 mg/L, no obvious decrease of the removal performance was observed. The reason might be that the seawater had a considerably high alkalinity. The pH measured in the experiment was about 7.8–8, and the pH of the system could be maintained above 7.5 during operation, so it would not have a great impact on microbial activity. In addition, under the condition of 300 mg/L alkalinity, it was found that the TOC effluent content was lower, which indicated that too much added inorganic carbon might cause the formation of a carbon residue in the system.

#### 3.2.2. Influence of Different Influent Alkalinity Values on Phosphate Removal Efficiency

**Figure 6:**
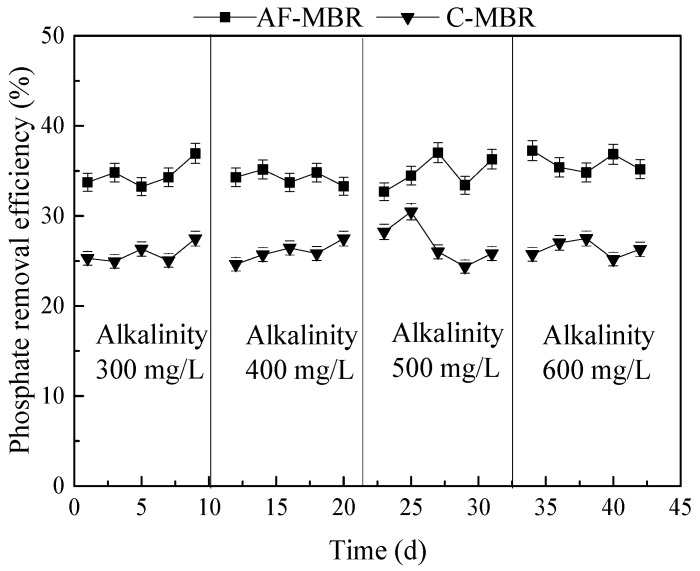
Influence of different influent alkalinity values on phosphate removal efficiency.

The treatment performance of AF-MBR and C-MBR in phosphate removal is shown in Figure 6. At present, many studies have focused on the effect of alkalinity on nitrogen removal, but few studies have analyzed the effect on phosphate removal, especially in a high-salt environment. The results of this experiment showed that the influent alkalinity had no obvious effect on the phosphate removal efficiency, and the phosphate removal efficiencies of AF-MBR and C-MBR were between 32.69% and 37.24% and between 24.36% and 30.47%, respectively. The treatment efficiency of AF-MBR was better than that of C-MBR. Because there were aerobic and anoxic sections in the AF-MBR, the phosphorus-accumulating bacteria could carry out a complete process of anaerobic phosphorus release and aerobic phosphorus absorption in the examined condition. Therefore, the treatment efficiency of AF-MBR was better than that of C-MBR.

Biological phosphorus removal was mainly achieved by removing phosphorus-rich sludge from the reaction system. However, in this experiment, the growth of microorganisms in the high-salinity environment would be limited to a certain extent, and the sludge discharge of the two groups of reactors was less, so the removal efficiencies were not high. During this process, alkalinity might change the pH of the solution and cause the formation of phosphate precipitates [45]. It was reported that the significant removal of phosphate from a solution occurred through the formation of phosphate precipitates, and the phosphate concentration was reduced to 24.8 mg/L [46]. However, in this study, the influent phosphate concentration was only about 10 mg/L, and the phosphate precipitates were difficult to form. Therefore, the influent alkalinity had no obvious effect on the phosphate removal efficiency. 

#### 3.2.3. Influence of Different Influent Alkalinity Values on Nitrogen Removal Efficiency

**Figure 7:**
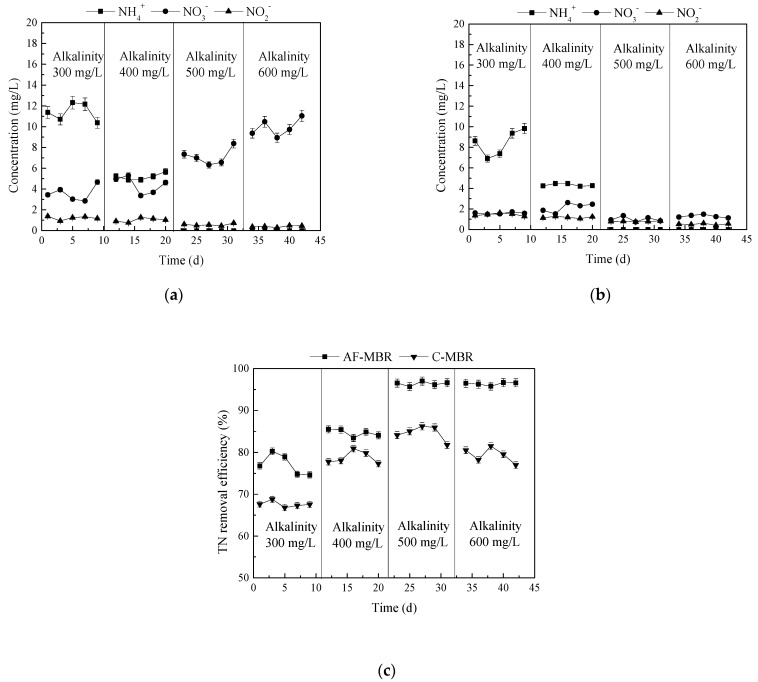
Effect of different influent alkalinity values on nitrogen treatment. (**a**) TN removal efficiency; (**b**) changes of NH_4_^+^-N, NO_2_^−^-N, and NO_3_^−^-N concentrations in AF-MBR; (**c**) changes of NH_4_^+^-N, NO_2_^−^-N, and NO_3_^−^-N concentrations in C-MBR.

In the biological process of nitrogen removal for wastewater, nitrification is a very important process that needs a certain amount of alkalinity, so the alkalinity of wastewater has a great impact on the effect of biological processes on nitrogen removal [47]. The changes of NH_4_^+^-N, NO_2_^−^-N, and NO_3_^−^-N in AF-MBR and C-MBR in different influent alkalinity conditions are shown in Figure 7a,b. As shown in Figure 7a, in C-MBR, in the case of low influent alkalinity, nitrification was limited, and the NH_4_^+^-N removal efficiency was low. In the case of high influent alkalinity, the nitrification reaction was completed, and denitrification had a great influence on NO_2_^−^-N and NO_3_^−^-N nitrogen accumulation. It can be seen in Figure 7b that, under a lower influent alkalinity, the concentration of NH_4_^+^-N was higher in the AF-MBR system due to restricted nitrification. With the increase of influent alkalinity, nitrification could be carried out completely, so the concentration of NH_4_^+^-N was reduced. Moreover, due to the backflow between the pre-anoxic filter and MBR in AF-MBR, the microorganisms could complete the nitrification and denitrification processes.

The changes of total nitrogen in the AF-MBR and C-MBR systems with changes in influent alkalinity are shown in Figure 7c. It can be seen in Figure 7c that, with the increase of influent alkalinity, the total nitrogen removal efficiencies of AF-MBR and C-MBR improved, but a change in the removal efficiency was not obvious when the alkalinity increased from 500 mg/L to 600 mg/L. In the whole process, the treatment efficiency of AF-MBR was always better than that of C-MBR.

The availability of inorganic carbon was critical for stable nitrification to provide sufficient alkalinity to buffer the acidification resulting from the production of hydrogen ions during ammonia oxidation. Furthermore, the loss of nitrification resulted in a decrease in nitrate concentrations being recycled back to the anoxic reactor, which led to reduced inorganic carbon formation through denitrification, further exacerbating the shortage of inorganic carbon in the aerobic cell [44]. The provision of adequate alkalinity for nitrogen removal was therefore essential for a stable treatment.

### 3.3. Effect of Different Ammonia Nitrogen Loads on AF-MBR Treatment Performance

Ammonia nitrogen is the main pollutant in marine aquaculture wastewater. In order to investigate the treatment efficiency of AF-MBR for simulated marine aquaculture wastewater with different ammonia nitrogen concentrations, the influent ammonia nitrogen concentrations of 20 mg/L, 30 mg/L, 40 mg/L, and 50 mg/L were chosen, with HRT of 8 h and influent alkalinity of 500 mg/L.

#### 3.3.1. Influence of Different Ammonia Nitrogen Loads on TOC Removal Efficiency

**Figure 8:**
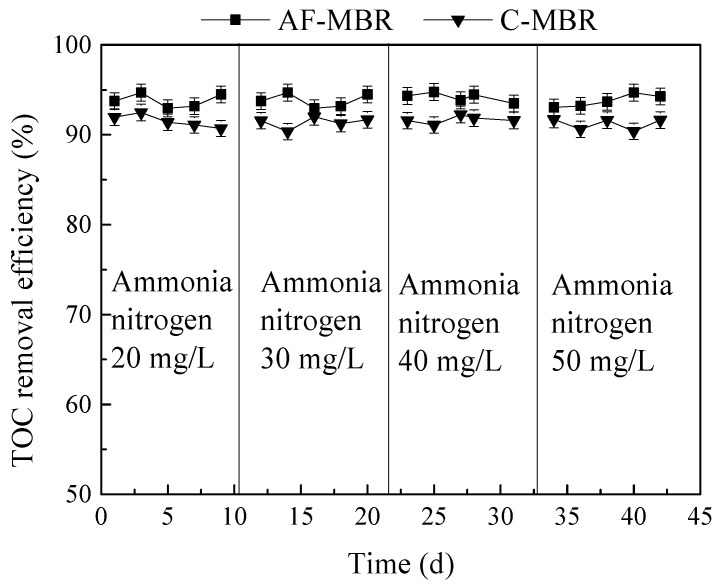
Influence of different ammonia nitrogen loads on TOC removal efficiency.

The TOC removal efficiency of AF-MBR and C-MBR under different influent ammonia nitrogen loads is shown in Figure 8. It can be seen from Figure 8 that when the influent ammonia nitrogen load changed from 20 mg/L to 50 mg/L, the TOC removal efficiency of C-MBR was basically maintained between 90.35 and 92.47%, while that of AF-MBR was between 92.95% and 94.75%. When the concentration of ammonia nitrogen changed in the range from 20 mg/L to 50 mg/L, the AF-MBR achieved a highly efficient removal of TOC. Through the experiments, it was found that the treatment efficiencies for TOC of AF-MBR and C-MBR were quite good. It was reported that organic pollutants removal of MBR was excellent (above 95%), suggesting that it was irrespective of the C/N ratio investigated, and the physical membrane enabled to further increase the removal efficiency of organic pollutants, achieving an almost complete removal [48]. The removal efficiency of AF-MBR for TOC was better than that of C-MBR. This was mainly due to the fact that the sludge used in the system had high biological activity, a stable population structure, and strong biodegradability and could quickly adapt to influent quality.

#### 3.3.2. Influence of Different Ammonia Nitrogen Loads on Phosphate Removal Efficiency

**Figure 9:**
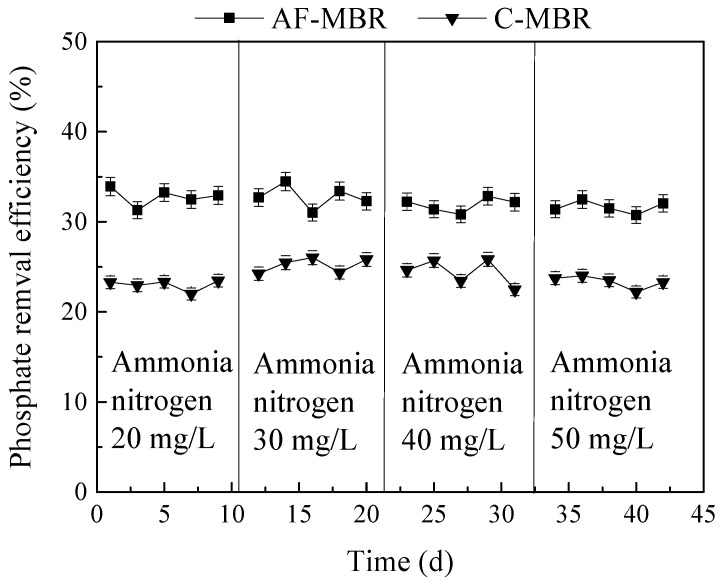
Influence of different ammonia nitrogen loads on phosphate removal efficiency.

The phosphate removal efficiency of AF-MBR and C-MBR is shown in Figure 9. It can be seen that when the concentration of ammonia nitrogen varied from 20 mg/L to 50 mg/L, the phosphate removal efficiency of the C-MBR fluctuated from 22.00% to 26.01%, while the phosphate removal efficiency of AF-MBR varied from 30.75% to 34.47%. Due to the limitation of sludge discharge, the phosphate removal efficiency of C-MBR was very low. In AF-MBR, the anoxic environment of the prefilter and the aerobic environment of the MBR provided the conditions of aerobic phosphorus uptake and anaerobic phosphorus release for phosphorus-accumulating bacteria. However, the growth of microorganisms is limited to a certain extent under high salt conditions. Even if phosphorus-accumulating bacteria could complete the whole process of phosphorus removal, the removal efficiency of phosphate was still not high. The phosphate removal efficiency was insignificantly affected by ammonia nitrogen concentrations between 20 mg/L and 50 mg/L.

#### 3.3.3. Influence of Different Ammonia Nitrogen Loads on Total Nitrogen Removal Efficiency

**Figure 10:**
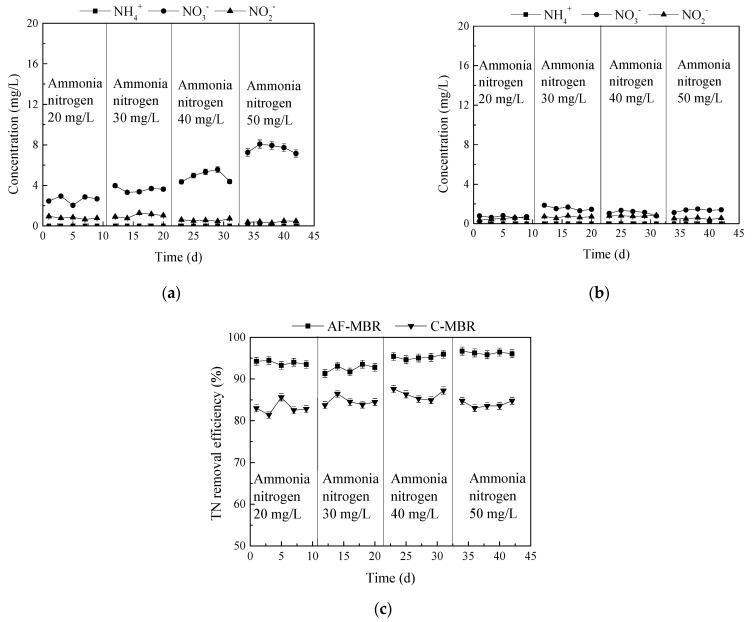
Effect of different ammonia nitrogen loads on nitrogen treatment. (**a**) TN removal efficiency; (**b**) changes of NH_4_^+^-N, NO_2_^−^-N, and NO_3_^−^-N concentrations in AF-MBR; (**c**) changes of NH_4_^+^-N, NO_2_^−^-N, and NO_3_^−^-N concentrations in C-MBR.

The changes of NH_4_^+^-N, NO_2_^−^-N, and NO_3_^−^-N in AF-MBR and C-MBR under different ammonia nitrogen loads are shown in Figure 10a,b. In C-MBR, with the continuous increase of influent ammonia nitrogen load, the concentration of nitrate nitrogen in the reactor also increased. Because the whole reactor was in the aerobic state and the sludge concentration in the reactor was relatively high, nitrifying bacteria could grow and reproduce. Therefore, the ammonia nitrogen in the influent was transformed into nitrate nitrogen and nitrite nitrogen under the action of nitrifying bacteria, and the concentration of nitrate nitrogen was higher than that of nitrite nitrogen due to the sufficient nitrification reaction.

With the continuous increase of influent ammonia nitrogen load, the concentrations of NH_4_^+^-N, NO_2_^−^-N, and NO_3_^−^-N in AF-MBR were at a relatively low level, and the concentration of NH_4_^+^-N was close to zero. With the continuous increase of ammonia nitrogen load, the concentrations of NO_2_^−^-N (2 mg/L) and NO_3_^−^-N (1 mg/L) increased little. AF-MBR can provide an anoxic environment for denitrifying bacteria, so the concentrations of NH_4_^+^-N, NO_2_^−^-N, and NO_3_^−^-N in AF-MBR were relatively low.

The removal of total nitrogen by AF-MBR and C-MBR under different influent ammonia nitrogen loads is shown in Figure 10c. It can be seen that when the concentration of ammonia nitrogen varied from 20 mg/L to 50 mg/L, the total nitrogen removal efficiency of C-MBR fluctuated from 81.40% to 87.60%, while the total nitrogen removal efficiency of AF-MBR varied from 91.30% to 96.68%.

NH_4_^+^-N loading plays an important role in nitrification [49]. The typical NH_4_^+^-N concentration in aquaculture wastewater is below 50 mg/L [50,51]. It was reported that the oxidation rates of ammonia and nitrite were reduced by 50% at NH_4_^+^-N concentrations up to 1 and 5 g/L [52]. Zhang et al. demonstrated the feasibility of increasing ammonium from 70 mg/L to 200 mg/L for the rapid start-up of a membrane bioreactor [53]. Usually, the MBR system is used to evaluate the efficiency of nutrient removal when treating synthetic high-strength water with a high concentration of nitrogen and phosphorus. However, in this study, the concentrations of nitrogen and phosphorus were relatively low. The MBR process itself has the characteristic of high biomass density, coupled with the filtration and interception function of the membrane module. When the influent concentration of ammonia nitrogen varied from 20 mg/L to 50 mg/L, the removal efficiencies of pollutants in the MBR were stable.

The removal efficiency of AF-MBR was more stable than that of C-MBR. For AF-MBR, the pre-anoxic biofilm unit played a good load buffer role, because the use of a carrier allowed a large number of microorganisms to adhere and grow in a biofilm, whose efficient adsorption and degradation ability ensured that the organic load in the subsequent MBR was maintained at a low level. Thus, this avoided the simultaneous entry of organic matter and ammonia nitrogen, which caused a competition between heterotrophic bacteria and nitrifying bacteria for dissolved oxygen or other nutrients, resulting in the decline of the nitrification efficiency. Therefore, the treatment efficiency of AF-MBR was better than that of the C-MBR.

## 4. Conclusions

In this study, the effects of different HRTs, different influent alkalinity values, and different ammonia nitrogen loads on the treatment of mariculture wastewater by AF-MBR were investigated. The reason for the better removal efficiency of TOC and total nitrogen at the HRT of 8 h is that a highly saline condition may inhibit microbial activity at a lower HRT, and microbial acclimatization to the saline environment of the bioreactor could recover the biological performance at longer HRTs. A long HRT with constant concentration of organic matter did not increase sludge concentration. With the increase of HRT, the phosphate removal efficiency decreased due to the limitation of sludge discharge. With the increase of influent alkalinity, the removal of TOC insignificantly changed. For phosphate removal, phosphate precipitates were difficult to form as a result of the low influent phosphate concentration. Therefore, the influent alkalinity had no obvious effect on phosphate removal efficiency. The total nitrogen removal efficiency increased with the alkalinity increasing from 300 mg/L to 500 mg/L. When the alkalinity changed from 500 mg/L to 600 mg/L, the change of total nitrogen removal efficiency was not obvious. The provision of adequate alkalinity for nitrogen removal is therefore essential for a stable treatment. The microorganisms could gather and grow on the carrier forming a stable biofilm with enhanced ability to resist a load impact. When the ammonia nitrogen load of the influent was changed from 20 mg/L to 50 mg/L, the TOC, phosphate, and total nitrogen removal efficiencies were relative stable.

## Data Availability

Not applicable.
